# Memory Macrophages

**DOI:** 10.3390/ijms24010038

**Published:** 2022-12-20

**Authors:** Malgorzata Kloc, Jacek Z. Kubiak, Robert Zdanowski, Rafik M. Ghobrial

**Affiliations:** 1 The Houston Methodist Research Institute, Transplant Immunology, Houston, TX 77030, USA; 2 Department of Surgery, The Houston Methodist Hospital, Houston, TX 77030, USA; 3 Department of Genetics, MD Anderson Cancer Center, The University of Texas, Houston, TX 77030, USA; 4 Dynamics and Mechanics of Epithelia Group, Faculty of Medicine, Institute of Genetics and Development of Rennes, University of Rennes, CNRS, UMR 6290, 35043 Rennes, France; 5 Laboratory of Molecular Oncology and Innovative Therapies, Military Institute of Medicine National Research Institute, Szaserow 128, 04-141 Warsaw, Poland

**Keywords:** macrophage, innate immunological memory, trained immunity, transplantation, epigenetic modifications

## Abstract

Immunological memory is a crucial part of the immune defense that allows organisms to respond against previously encountered pathogens or other harmful factors. Immunological memory is based on the establishment of epigenetic modifications of the genome. The ability to memorize encounters with pathogens and other harmful factors and mount enhanced defense upon subsequent encounters is an evolutionarily ancient mechanism operating in all animals and plants. However, the term immunological memory is usually restricted to the organisms (invertebrates and vertebrates) possessing the immune system. The mammalian immune system, with innate and adaptive branches, is the most sophisticated among vertebrates. The concept of innate memory and memory macrophages is relatively new and thus understudied. We introduce the concept of immunological memory and describe types of memory in different species and their evolutionary status. We discuss why the traditional view of innate immune cells as the first-line defenders is too restrictive and how the innate immune cells can accumulate and retain immunologic memory. We describe how the initial priming leads to chromatin remodeling and epigenetic changes, which allow memory macrophage formation. We also summarize what is currently known about the mechanisms underlying development of memory macrophages; their molecular and metabolic signature and surface markers; and how they may contribute to immune defense, diseases, and organ transplantation.

## 1. What Is Immunological Memory?

Immunological memory is an evolutionary adaptation of the immune system. It mediates faster and enhanced responses to previously encountered antigens through the ability of immune cells to remember encountered pathogens or other immunogenic factors [1,2]. An obvious prerequisite for immunological memory is that the remembering cells should be long-lived and maintain memory in the absence of the original factor that induced memory. A well-known example of immunological memory is long-term protection from an infection acquired after one-time vaccination or a single infection. Studies showed that the number of memory cells specific to a given factor remains stable, indicating a strict control of memory cell proliferation and death [1,3,4]. The ability of organisms to memorize encounters with a pathogen or another harmful factor and mount a faster and stronger defense upon subsequent encounter is probably an evolutionary ancient adaptive trait because it occurs in bacteria, animals (invertebrates and vertebrates), and plants [5,6,7,8]. However, the term immunological memory is usually restricted to the animals (invertebrates and vertebrates) possessing the immune system. In vertebrates, immunological memory probably evolved twice. First, as a variable lymphocyte receptor (VLR)-based memory of lymphocyte-like cells in the jawless vertebrates, and second, as the immunoglobulin (Ig)-based memory in other vertebrates [5,9]. Besides Crispr/Cas-based memory in bacteria and archaea [10], a recently introduced multidimensional model across different species recognizes five different types of memory. 1. Systemic acquired resistance (SAR) in plants [7]. 2. Priming (IP) and transgenerational immune priming (TGIP), i.e., vertical transmission of immune experience from parents to the offspring; horizontal transfer between individuals, and between individuals and other parents’ offspring, in insects and other invertebrates [11,12]. 3. NK-cell immune memory [13]. 4. Myeloid cell (monocytes, macrophages, and dendritic cells) nonspecific memory, sometimes also referred to as trained innate immunity (TII) or trained memory [14,15]. 5. Classical adaptive memory in vertebrates [2].

## 2. Memory Macrophages

Although there are many studies and descriptions of memory T and B cells [16,17,18,19], the concept of innate memory and memory macrophages was formulated only a decade ago [20]. Such cunctation was a consequence of the traditional division between innate and adaptive (acquired) immunity, where the innate immune cells, such as macrophages, were always considered with the first line of defense against invading pathogens and lacking adaptive abilities. According to this view, the only role of innate immune cells is to prevent pathogen entry and rapid elimination of internalized pathogens before they can cause illness. Additionally, in vertebrates, the innate immune cells also process and present antigens to the adaptive immune cells. Elimination of pathogens by innate immune cells is an evolutionarily ancient mechanism of defense. It is nonspecific and relies on the recognition of pathogen-associated molecular patterns (PAMPs), which are molecules frequent in many different microbes, by pattern recognition receptors of the immune cells (PRRs) [21,22,23,24]. Some examples of PAMPs are lipopolysaccharide (LPS) and porins of Gram-negative bacteria; peptidoglycans of Gram-positive bacteria; flagellin from bacterial flagella; β–glucans and mannans from fungi; and bacterial and viral nucleic acids. Although different PAMPs are chemically and structurally unrelated they share features that allowed the evolution of the immune recognition strategy. First, all these factors are produced by microbes but not by the host, which prevents immune response against the self. Second, they are shared by a given class of microbes, which allows a limited variety of PRRs to recognize any microbe. Third, PAMPs are usually the molecules essential for microbe survival and thus cannot frequently mutate to avoid PRR recognition without a lethal consequence.

Recent studies show that the traditional view of innate immune cells, that is to say as limited to the first line of defense, is too restrictive and that the innate immune cells can also accumulate and retain immunologic memory [15]. The classical paradigm postulates the innate-to-adaptive directionality of immunological memory formation. In this scenario, after encountering the antigens the innate immune cells, besides presenting processed antigens to B cells and T cells, produce mediators and signals such as IL-12 which trigger the development of memory T cells. However, recent studies indicate that there is also adaptive-to-innate directionality of memory formation. In this scenario, shown in the respiratory adenoviral infection model, the T cells activated by the specific antigen induce the development of memory macrophages via the IFN-γ pathway [25]. These findings led to redefining immunological memory as the feature of both innate and adaptive immune systems [26].

Both innate and adaptive memory development has two main steps. Initial priming (activation) is followed by the phase of the immune response. Immunological memory is the ability to mount a robust and more effective (potentiated) response to secondary exposure to identical or similar antigens/pathogens. The main differences between innate and adaptive memory are their longevity and specificity. While the innate memory persists for months [25,27,28] and lacks antigen specificity, the adaptive memory is lifetime or long-lived and specific for a given antigen [29,30]. The memory macrophages can be both systemic and tissue specific. Studies from systemic infection or immunization show that these processes induce memory in circulating monocytes and macrophages. The development of systemic innate memory depends on the trained myeloid progenitors in the bone marrow [31,32,33]. In other cases, such as lung infection, the memory macrophages derive from the pool of resident macrophages established in the embryo. When these memory macrophages die, they are replenished from a pool of circulating monocytes, which settle in the tissue and enter the memory-establishing pathway [25]. Netea et al. [5] suggested that the innate immune memory, which is evolutionarily more primitive because of its non-specific response to reinfection, relies mainly on the rearrangement of chromatin structure. The adaptive immune memory, which is evolutionarily younger and functionally more advanced, relies on epigenetic changes and genetic recombination-derived specificity. However, studies of the last decade also indicate that innate memory heavily depends on epigenetic changes, which often influence chromatin structure and compaction [34,35]. Epigenetic modifications rely mainly on three mechanisms: methylation of DNA, modification of histones, and regulation of gene expression by non-coding RNAs (ncRNAs) [36,37]. Epigenetic modifications of chromosomes alter gene expression, and although they do not change the DNA sequence, sometimes they are heritable. These so-called epigenetic tags can pass not only to consecutive generations of cells but also from parents to progeny [38,39]. The concept of inheritance of non-DNA-based alterations of the genome is unconventional and has a profound impact on our understanding of evolution. Conventional understanding has always been that, during fertilization, the fusing gametes undergo reprogramming, including erasing all epigenetic tags, which return their genomes to the “tabula rasa” state. However, around 1% of epigenetic tags escape reprograming through the imprinting process, which occurs in animals and plants. Therefore, the epigenetic modification inherited from a cell to its descendant or from a parent to its offspring may influence gene expression in the progeny [38,39,40,41,42]. Although there are indications that parents’ experience can affect the immune response of the progeny, additional research is necessary to prove that epigenetic modifications in the parental genomes affect transcriptional programs in subsequent generations. One of the possibilities is that the maternal information of the oocyte/egg affects the epigenetic program of the primordial germ cells. In contrast to mammals, where the primordial germ cells are induced in the embryonic epiblast before migrating to the nascent gonads, in many vertebrates and invertebrates, the primordial germ cell fate is already established in the oocyte. In some of these animals, such as *Xenopus*, oocytes contain a germ cell determinant called the germ plasm (containing RNAs and proteins), which segregates to the primordial germ cells. Thus, the primordial germ cells of the early embryo are already pre-programmed by the maternal information in the oocyte. One can imagine that in these animals, the epigenetic program of the mother can easily influence the progeny.

A fascinating finding pertaining to innate memory is that macrophage precursors, the hematopoietic stem cells (HSCs), not only respond to the infection through pattern-recognition receptors (PRRs) but also become epigenetically reprogramed and transmit this epigenetic memory to the daughter macrophages. Thus, the macrophages derived from trained HSCs are already epigenetically programmed for a robust transcriptional response against infection [43].

Another level of immune memory complexity derives from the recent discovery of the tissue imprinting phenomenon. Epigenomic, single-cell transcriptome, and fate mapping studies show that the niche where the immune cells reside can rewire or override their transcriptional program and imprint different transcriptional and metabolic identities [44].

Unfortunately, under certain circumstances, such programmed cells can be malefic and induce inflammatory pathologies and promote cancer development [45].

## 3. Chromatin Remodeling and Epigenetic Changes Induced by Initial Priming of Myeloid Cells

Gene expression depends on the accessibility of DNA in the promoters and enhancers regions to the transcription factors and RNA polymerases. DNA accessibility is regulated by chromatin compaction, DNA methylation, histone modifications (acetylation, methylation, phosphorylation, and citrullination), and immune-gene-priming long noncoding RNAs, such as the upstream master lncRNA of the inflammatory chemokine locus (UMILILO) [35,46,47,48,49]. In general, hypermethylation of DNA silences and hypomethylation induces gene transcription [50,51]. Modifications of the histones affect histone–histone and histone–DNA interactions, histone binding to chaperones, and chromatin structure [52]. Histone methylation results in transcriptional silencing or activation depending on the number of methyl groups added and which of a given histone is methylated [53]. For example, the trimethylation of lysine 4 at histone 3 (H3K4me3) and H3K4me1 activates promoters and enhancers, respectively [54].

In unstimulated macrophages/myeloid cells, the regions of chromatin housing the pro-inflammatory genes are compacted and unavailable to the transcription machinery. The primary stimulation with the antigen/pathogen recruits various transcription factors, such as activator protein 1 AP-1; the signal transducers and activators of transcription STATs; and nuclear factor-kappa B (NF-kB) to the promoters and enhancers, which are already pre-marked in the naïve cells by the lineage-specific PU.1 transcription factor [5,55]. The PU.1 transcription factor, which belongs to the ETS-domain transcription factor family, is specific for myeloid and B-lymphoid cells [56]. Transcription factors recruited to the PU.1-marked regions conscript chromatin remodelers and histone acetyltransferase. These, in turn, increase acetylation and change chromatin conformation permitting the recruitment of RNA polymerase II [5,57]. Histone acetylation activates both promoters and enhancers of pro-inflammatory genes. For example, acetylated Lys56 of H3 (H3K56) affects nucleosome-DNA interactions and chromatin structure and compaction, while acetylation of Lys91 of histone H4 leads to nucleosome instability [52].

**Figure 1:**
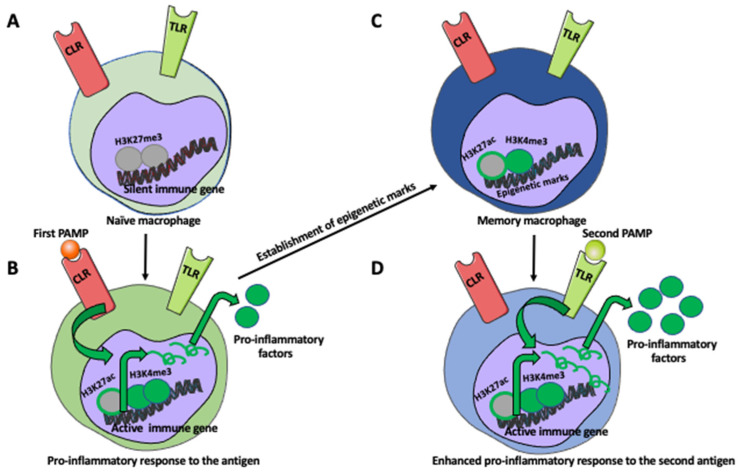
Development of memory macrophage.(**A**) Naïve (unstimulated) macrophage expresses pattern recognition receptors PRSs, such as Toll-like receptors (TLRs) and non-TLR antigen-recognition receptors, such as C-type lectin receptors (CLRs), which recognize pathogen-associated molecular patterns (PAMPs). For example, TLR-4 recognizes lipopolysaccharide (LPS) of Gram-negative bacteria, and CLR receptor Dectin-1 recognizes β-glucans present in bacteria and fungi. In the naive macrophage, the pro-inflammatory genes are transcriptionally silent via, for example, the trimethylated H3K27 (H3K27me3). (**B**) The binding of PAMs molecule to its receptor induces histone modifications at the gene promoter, such as trimethylation of H3K4 (H3K4me3) and acetylation of H3K27 (H3K27ac). In addition, the enhancer region becomes enriched with monomethylated H3K4 (H3K4me1) and H3K27ac. Activated pro-inflammatory genes transcribe mRNAs, which after transport to the cytoplasm, are translated into the pro-inflammatory cytokines, such as IL-6 and TNF-α, which fight the pathogen. (**C**) After pathogen elimination, some histone modifications in the macrophage are lost, but some remain as the epigenetic marks. The immunological memory of the first encounter with the pathogen becomes imprinted in the macrophage genome. (**D**) The state of transcriptional alertness of memory macrophage allows for faster and stronger pro-inflammatory response upon a second encounter with the same or different pathogens.

**Figure 2:**
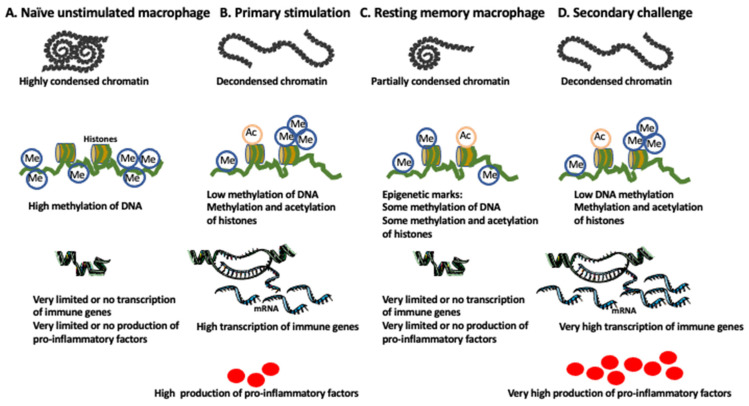
Chromatin structure and epigenetic marks during the development of memory macrophages. (**A**) In naïve (unstimulated) macrophages, the regions of chromatin housing the immune response genes are highly condensed (heterochromatin state) due to high methylation of DNA, making these genes inaccessible to the transcription factors. This results in complete silencing or very low transcription and translation of the immune response genes. (**B**) Primary stimulation with the pathogen or danger signals demethylates DNA, decondenses chromatin (euchromatin state), and methylates and acetylates histones (for example, H3K4me3, H3K27ac) reading the immune gene for transcription. A high level of transcription followed by translation produces a high level of immune response factors. (**C**) After cessation of the stimulus, chromatin only partially condenses, and the remaining epigenetic marks (for example, H3K4me1) keep resting memory macrophage in the state of transcriptional alertness. (**D**) The secondary challenge, with the same or different pathogen, induces chromatin decondensation, demethylation of DNA, and modification of histones (such as H3K4me3, H3K4me1, and H3K27ac) allowing robust transcription and subsequent high production of immune response factors. Modified from [54].

After cessation of the stimulus, some of these histone modifications remain, thus allowing fast and efficient transcription of the pro-inflammatory genes upon restimulation [5,58]. Studies showed that some of the enhancers, called latent enhancers, are not pre-marked in naïve cells but acquire histone modifications upon primary stimulation. After the removal of the stimulus, some of these latent enhancers also retain histone modifications, which permits faster and stronger activation upon restimulation (Figure 1 and Figure 2) [5,59,60].

## 4. Molecular Signature and Markers of Memory Monocytes/Macrophages

The epigenetic markers of memory monocytes consist of increased methylation and acetylation of histones (H3K4me1, H3K9me2, H3K4me3, and H3K27Ac) on the promoters of genes coding for immune defense and metabolism factors [53,61,62]. For example, the Bacillus Calmette–Guérin (BCG) vaccine-trained macrophages have increased H3K4 trimethylation on the promoter of IL-6 and TNF [61]. Exposure of monocytes/macrophages to microbes upregulates synthesis of pathogen-associated molecular patterns (PAMPs) and damage-associated molecular patterns (DAMPs) receptors, such as dead cell engulfment receptor Draper (*Drosophila melanogaster* homolog of the Ced-1 protein of *Caenorhabditis elegans*), which increase affinity to the pathogen, damaged cells, and tissues [63,64]. Exposure of macrophages to *Staphylococcus aureus* induced expression of IL-6 and IL-17A on day 2 and day 7, respectively. Interestingly, the memory developed by macrophages after *S. aureus* skin infection is skin-specific and can be transferred to the naïve individuals [20].

Studies of human monocytes showed that short exposure to bacteria, the components of bacterial walls, or vaccines induced a proinflammatory phenotype, which lasted over three months and consisted of robust production of inflammatory factors such as Il-6 and TNF-α [53,65,66]. Another example of innate memory is the immune memory of alveolar macrophages that persists at least four months after the initial viral exposure [26,28]. Alveolar macrophages derive from hemopoietic progenitors, which settle in the developing lungs before birth. Owing to the fact that alveolar macrophages are self-propagating, long-lived, and do not rely on replenishment by circulating monocytes, they are very well suited to developing immunologic memory. Studies showed that adenovirus infection induces alveolar macrophage memory, which protects against subsequent bacterial infections. These memory macrophages increase glycolysis and upregulate pro-inflammatory genes only at the transcriptional level. By refraining from producing pro-inflammatory cytokines until they encounter infecting bacteria, alveolar memory macrophages can keep uninfected lungs in an inflammation-free state [26,27,67]. Yao et al. [25] studied surface markers of alveolar memory macrophages. In addition to the markers commonly found in many macrophage subtypes, such as a high level of integrin alfa X (CD11c), integral membrane glycoprotein Fc receptor (CD64), and sialic-acid-binding immunoglobulin-like lectin (Siglec-F), the memory lung macrophages expressed high levels of MHC II. They also expressed the mannose receptor (CD206), toll receptors (TLR2/4), and integrin alfa M (CD11b), but the level of these markers did not increase after activation with the virus. The development of alveolar memory macrophages is also interwoven with the epigenetic modification of histones [49]. Interestingly, frequent episodes of lung inflammation deplete resident memory macrophages allowing the recruitment of inflammatory monocytes, which after a few days, develop into a new generation of resident memory macrophages [68]. One of the important factors affecting epigenetic changes in macrophages is chronic stimulation of their innate immune receptors such as the nucleotide-binding oligomerization domain 2 (NOD2) or multiple PRRs. NOD2 is an intracellular sensor for small peptides derived from the peptidoglycan of the bacterial cell wall. Studies showed that while primary activation of the NOD2 increases acetylation of H3 and H4 cytokine promoters, chronic stimulation decreases acetylation. Long-term stimulation of NOD2 stimulation activated Twist1 and Twist2 transcription factors, induced the expression of histone deacetylase HDAC1 and HDAC3 by binding to their promoters. Consequently, HDAC1 and HDAC3 deacetylated histones at the cytokine promoters and downregulated H3 and H4 cytokines [69].

It is known that energy metabolism and dietary compounds affect enzymes modulating chromatin compaction and structure [60,70]. Thus the metabolites, through epigenetic changes, also affect macrophage immune memory. For example, ATP, NAD (+), acetyl-CoA, and S-Adenosyl methionine (SAM), regulate epigenetic enzymes responsible for nucleosome distribution, DNA methylation, and modifications of histones [60,71].

One of the molecular markers described in memory monocytes/macrophages primed with β-glucan (from the cell wall of *Candida albicans* yeast) is a metabolic shift from oxidative phosphorylation to aerobic glycolysis. These cells have a high glucose consumption, upregulate lactate synthesis, and have a high NAD (+)/NADH ratio. This metabolic shift depends on β-glucan receptor dectin-1, RAC (Rho family)-alpha serine/threonine-protein kinase Akt, oxygen homeostasis regulator hypoxia-inducible transcription factor-1α (HIF-1α), and mammalian target of rapamycin (mTOR) pathways [53,72,73]. Arts et al. [74] also showed that the BCG-trained monocytes upregulated glycolysis. The inhibition of glycolysis enzymes affected histone methylation and prevented the development of immune memory. Studies of human monocytes trained by exposure to β-glucan showed that they accumulate fumarate (a metabolite of tricarboxylic acid cycle (TCA)), which downregulates histone demethylase KDM5. Another TCA metabolite, the α- ketoglutarate, mediates epigenetic reprograming through the histone demethylase Jumonji domain-containing protein-3 (JMJD3) that regulates trimethylation of histone H3 on lysine 27 [60,75].

Interestingly, the metabolic shift in macrophages does not always lead to the development of pro-inflammatory memory but in some instances, preprograms macrophages toward anti-inflammatory responses. Studies by Du et al. [76] in the autoimmune encephalomyelitis model showed that human mesenchymal stem cells (MSCs) preconditioned under low oxygen (LO-MSCs) secrete insulin-like growth factor 2 (IGF-2) that shifts the metabolism of maturating macrophages (but not the mature macrophages) from glycolysis toward persistent oxidative phosphorylation (OXPHOS) and anti-inflammatory phenotype. The subsequent pro-inflammatory challenge was unable to reverse this metabolic commitment, and the macrophages remained in a permanent anti-inflammatory state. Epigenomic analysis showed that IGF-2 pre-programming changed the distribution of H3K27ac in the promoters and enhancers of macrophages. Reduced H3K27ac was observed in genes critical for pro-inflammatory responses, and increased H3K27ac in the anti-inflammatory genes.

## 5. Memory Macrophages in Transplantation

Epigenetically driven transcriptional readiness of memory macrophages to respond to a nonspecific secondary challenge poses a potential problem of the hyperreactive immune response against transplanted organs. The immune response to the graft consists of two phases: the first phase of a rapid response by the innate immune cells (macrophages, dendritic cells, and neutrophils) is followed by a slower, adaptive immune cell (T cells and B cells) response. All currently existing anti-rejection therapies ignore the role of innate cells and focus on the subduing adaptive immune response [73,77]. Macrophages are crucial players in both acute and chronic allograft rejection [78,79,80]. They produce pro-inflammatory cytokines and costimulatory molecules, such as CD80 and CD86. The inflammatory cytokines recruit T cells while costimulatory molecules binding to their appropriate ligands expressed on T cells mobilize T cell responses against the graft. Transplanted organs injured by ischemia-reperfusion release damage-associated molecular patterns (DAMPs) molecules, which bind to the pattern recognition receptors of non-trained and memory (trained) macrophages [77]. We postulate that the immune response of untrained macrophages to DAMPs is relatively slow and meager. In contrast, the memory macrophages, which are already epigenetically programmed for a rapid and robust immune response, produce very high quantities of inflammatory cytokines and strongly activate the adaptive immune system, resulting in vigorous rejection of the transplant. Given that the immune response of memory macrophages lasts in humans for many months [66], the hyperreactive response of memory macrophages will exacerbate graft rejection. Although there are no data on memory macrophages in human transplantation, Ochando et al. [77] suggested that the main pathways involved in the development of memory macrophages and contributing to graft rejections are cell death, microbial infection, and oxidation of lipids.

(1) The ischemia-reperfusion injury of the graft generates a massive number of dying cells. Danger-signaling molecules exposed or released from the apoptotic and necrotic cells are, for example, vimentin and high mobility group box 1 HMGB1 protein (which belongs to the DAMP family). These molecules will bind to their respective macrophage receptors, Dectin-1 (a transmembrane pattern-recognition receptor specific for β-glucan carbohydrates) and toll-like receptor 4 (TLR4), stimulating the expression of pro-inflammatory cytokines, inducing epigenetic modifications, and generating macrophage memory [77,81]. Studies in a mouse transplantation model support this model. They showed that the production of inflammatory cytokines by memory macrophages stimulated by TLR4 and Dectin-1 is driven by epigenetic modifications and aerobic glycolysis [82,83]. During many months of existence, the memory macrophages created in response to the danger signals from dying cells will mount a hyperactive immune response against the graft.
(2) Many studies showed that viral or bacterial infection exacerbates graft rejection [73,84]. It is plausible that memory macrophages produced in response to infection mount an enhanced immune response against the graft. The molecules and pathways involved in infection-induced memory are NOD2; possibly viral RNA; and NOD-, LRR-, and pyrin domain-containing protein 3 (NRLP3), which is an intracellular sensor that detects a broad range of microbial molecules [69,77,85,86,87]. Studies showed that the Bacillus Calmette–Guérin (BCG) vaccine against tuberculosis induces NOD2-dependent protection against secondary infections through epigenetic reprogramming of monocytes/ macrophages. In the resulting memory macrophages, the promoters of IL-6 and TNFα genes had increased trimethylation of histone H3 at lysine (H3K4me3) and increased production of relevant cytokines [65].
(3) Another pathway possibly involved in the generation of memory macrophages and relevant to transplantation is the immune response induced by hyperlipidemia and oxidized low-density lipoprotein (oxLDL) [7]. Studies showed that accumulation of oxLDL within the graft is associated with the presence of macrophages, development of interstitial fibrosis, and graft failure [88]. The oxLDL belongs to DAMP. The binding of oxLDL to the CD36 receptor expressed in macrophages and other myeloid cells switches glycolysis, increases the production of pro-inflammatory factors, and induces innate cell memory [89].

## 6. Immune Tolerance Memory

Immune memory does not always lead to hyper-responsiveness and enhanced host defense. Under certain circumstances, it can lead to hypo-responsiveness and the development of immune tolerance memory toward the second microbial challenge. Immune memory tolerance develops when the macrophages are reputedly, or for a long time, exposed to the pathogen or its components. These macrophages minimize their responses, such as cytokine production, or cease responding to the subsequent exposure to the same or different pathogen. One of the examples is the development of long-lasting (weeks-months), and often fatal, immune tolerance memory toward bacterial infection of the lungs after clearance of previous influenza or syncytial respiratory virus infection. Studies showed that the virus desensitizes Toll-like receptors (sensors of bacterial flagellin) and lectin and mannose receptors. It also downregulates NF-κB signaling in alveolar macrophages. This results in lower responsiveness toward bacterial proteins and lowered expression of pro-inflammatory factors TNF-α and IL-17. The IFN-α/β, IFN-γ, and Il-10 produced during viral infection further lower anti-bacterial responses of macrophages by inhibiting the production of oxyradicals [26,90,91,92]. Studies showed that monocyte/macrophage immune tolerance memory depends on the failure to accumulate epigenetic active histone tags at the promoter and enhancers of the anti-bacterial pathway’s genes. They also showed that β-glucan reinstates cytokine production and partially reverses macrophage immune tolerance. The reactivation of transcriptionally silent genes depends on the reinstatement of epigenetic (histone modifications) tags [93]. In the context of transplantation, studies in mice heart and kidney transplantation models showed that infection with the parasite *Trichinella spiralis* prevents graft rejection [84,94]. Although these studies concentrated on the effect on T cells and suppression of Th1/Th17 responses on the graft survival, it is possible that parasite infection also generated memory macrophages with suppressed proinflammatory responses.

## 7. Possible Avenues for Therapeutic Applications of Memory Macrophages

As in transplantation, most of the existing immune therapies target the adaptive immune system. However, an increasing knowledge of the innate immune memory opens new therapeutic possibilities. One such option is preventing the development of memory macrophages or reprogramming their memory. This could be achieved by inhibiting glycolysis or/and suppressing histone and DNA modifications (methylation) using various inhibitors. The induction of memory macrophages can involve the manipulation of pathways and receptors involved in the recognition of various pathogens.

## 8. Conclusions

Although the existence of innate immune cell memory is undeniable, the knowledge of mechanisms regulating its development and the consequences are still fragmentary. Further research is needed to define the molecular and cellular triggers of the epigenetic modifications in macrophages. Very little is still known about how the macrophage niche and tissue of residence affect memory macrophages. We also need to explore how innate memory cells contribute to various diseases and if they could be a valid therapeutic target. Very little is also known about how innate memory cells affect transplanted organs and acute and chronic rejection. Another fascinating and understudied subject is the inheritance of innate memory, and how it may affect homeostasis and response to diseases in the progeny.

## Data Availability

Not applicable.
